# Urine miRNA signature as a potential non-invasive diagnostic and prognostic biomarker in cervical cancer

**DOI:** 10.1038/s41598-021-89388-w

**Published:** 2021-05-14

**Authors:** Mehreen Aftab, Satish S. Poojary, Vaishnavi Seshan, Sachin Kumar, Pallavi Agarwal, Simran Tandon, Vijay Zutshi, Bhudev C. Das

**Affiliations:** 1 Amity Institute of Molecular Medicine & Stem Cell Research (AIMMSCR), Amity University Campus, Sector-125, Noida, Uttar Pradesh 201313 India; 2 Department of Gynecology and Obstetrics, Safdarjung Hospital, New Delhi, 110029 India; 3 Depatment of Medical Oncology, Dr. B R Ambedkar Institute Rotary Cancer Hospital, All India Institute of Medical Sciences, Ansari Nagar, New Delhi, 110029 India

**Keywords:** Biotechnology, Cancer, Computational biology and bioinformatics, Molecular biology

## Abstract

MicroRNAs as cancer biomarkers in serum, plasma, and other body fluids are often used but analysis of miRNA in urine is limited. We investigated the expression of selected miRNAs in the paired urine, serum, cervical scrape, and tumor tissue specimens from the women with cervical precancer and cancer with a view to identify if urine miRNAs could be used as reliable non-invasive biomarkers for an early diagnosis and prognosis of cervical cancer. Expression of three oncomiRs (miR-21, miR-199a, and miR-155-5p) and three tumor suppressors (miR-34a, miR-145, and miR-218) as selected by database search in cervical pre-cancer, cancer, and normal controls including cervical cancer cell lines were analyzed using qRT-PCR. The expression of miRNAs was correlated with various clinicopathological parameters, including HPV infection and survival outcome. We observed a significant overexpression of the oncomiRs and the downregulation of tumor suppressor miRNAs. A combination of miR-145-5p, miR-218-5p, and miR-34a-5p in urine yielded 100% sensitivity and 92.8% specificity in distinguishing precancer and cancer patients from healthy controls and it well correlates with those of serum and tumor tissues. The expression of miR-34a-5p and miR-218-5p were found to be independent prognostic factors for the overall survival of cervical cancer patients. We conclude that the evaluation of the above specific miRNA expression in non-invasive urine samples may serve as a reliable biomarker for early detection and prognosis of cervical cancer.

## Introduction

Cervical cancer is the fourth most common cancer in women with an estimated 300,000 deaths and 600,000 new cases reported in 2018^1^. Vast majority (~ 85%) of cervical cancer cases are detected in the less developed nations, where it accounts for ~ 12% of all female cancers^2^. Biologically, cervical cancer begins in the form of pre-malignant lesions referred to as squamous intraepithelial lesions (SILs) or cervical intraepithelial lesions (CINs) or dysplastic lesions that progress to carcinoma-in-situ to invasive cancer through different stages in a period of 10 to 20 years^3^. Persistent infection of high-risk (HR) human papillomaviruses (HPVs) has been considered as the principal cause for cervical cancer^4^. Currently, most commonly available HPV vaccines (Cervarix and Gardasil) provide protection against only two most common HR-HPV types, -16 and -18 including HPV 9—valent vaccine HPVs but not to other HR-HPV types^5,6^. Available evidence suggests that there is a long gap between HPV infection and development of low-grade lesions that progress to invasive cancer^7,8^. This window period provides a unique opportunity for understanding molecular pathogenic pathways involved in cervical tumorigenesis and identification of cancer biomarker(s) for early diagnosis and prognosis which may result in the better management and control of cervical cancer.

MicroRNAs (miRNAs) are small, non-protein coding RNAs of 18–25 nucleotides involved in post-transcriptional gene silencing by binding to 3′ untranslated region (UTR) of its target messenger RNAs (mRNAs) or degradation of homologous miRNAs. Since miRNAs are known to regulate diverse cellular and biological processes, including gene expression, cell proliferation, differentiation, inflammation, immune modulation, and programmed cell death, any deregulation in their expression may results in the initiation and progression of cancer. Overexpressed miRNAs may function as oncogenes by negatively regulating the expression of tumor suppressor genes, while downregulated miRNAs function as tumor suppressors by negatively regulating the oncogenic mRNAs. Differential expression of oncogenic and tumor suppressor miRNAs has been proposed as a candidate biomarker in many cancers^9,10^_._ Apart from their detection in tumor tissues, several studies have also delineated the role of miRNAs derived from liquid biopsy samples, including blood plasma and serum, in cervical cancer^11–14^. However, it is thought that urine could be a better non-invasive liquid biopsy sample than blood because of the homeostasis mechanisms inside the body which may reflect changes in the blood, hence expression of miRNAs^15^. Also, urine can be easily self-collected recurrently, in relatively large volumes. Although most studies have focused on the identification of biomarkers in blood, the ease of availability of urine and the high rate of patient compliance suggest that it could provide a promising source for the screening of patients for cervical cancer if the results match with that of the blood or tumor tissue. Since uterine cervical epithelial cells are continuously shading down in the urine, it has been demonstrated earlier that HPV can be detected in urine with high sensitivity and specificity^16–18^. However, at present, the studies on expression profiling of miRNAs in urine for their use as potential biomarker(s) for screening, early detection, and prognosis of cervical cancer are lacking. Therefore, early diagnosis and grade*/*stage-specific detection of cervical cancer using non-invasive self-urine sampling could pave the way for large scale screening studies in a controlled manner. Thus, the present study was designed to profile selected miRNA expression in urine and to compare the same with those of paired serum, cervical scrapes (in precancer cases) or cervical tumor tissues in order to validate specific urine miRNA(s) as reliable biomarkers. After a comprehensive literature survey and complete search of databases on these miRNA targets related to cervical cancer, we shortlisted a panel of six miRNAs—three oncomiRs (miR-199a-5p, miR-21-5p, miR-155-5p) and three tumor suppressors (miR-145-5p, miR-34a-5p, and miR-218-5p) to evaluate their functional role in HPV-induced cervical cancer.

## Results

### Prevalence of HPV infection in urine compared with paired cervical scrapes and biopsies in cervical pre-cancer, cancer and control

A noninvasive urine sampling has been utilized to establish if urine can serve as an alternative clinical material for reliable detection of HPV and other sensitive biomarkers such as miRNA expression for early detection of cervical cancer. Therefore, in the present study, paired urine samples, cervical scrapes and tissue biopsies from 50 subjects each of pre-cancer, cancer along with adjacent normal tissues and normal subjects were collected and subjected to detection and genotyping of HPV types 16 and 18 which are the most common high-risk oncogenic HPV types worldwide and are present in over 90% of carcinomas of Indian women. The analysis of HPV infection and its genotyping was conducted by PCR using consensus and HPV type-specific primers.

PCR-based detection revealed that 30 (60%) out of a total of 50 urine samples of pre-cancer lesions were HPV positive. Out of these 50 precancer urine samples, 17 LSIL and 33 HSIL had 4 (8%) and 26 (52%) HPV positives, respectively. HPV infection was detected in the cervical scrape of 35/50 (70%) of precancer lesions with 14% of LSIL and 56% of HSIL found to be HPV positive. However, in control samples (n = 50), only 3 (6%) urine samples and 4 (8%) normal cervical samples were found to be HPV positive (Table 1). Subsequently, HPV type specific PCRs performed in these cases to determine the prevalence of two most prevalent HR-HPV types 16 and 18 in urine samples revealed the presence of HPV16 DNA sequence in 28 out of 50 (56%) cervical pre-cancer cases [4(8%) of LSIL and 24 (48%) of HSIL] whereas in cervical scrapes it was 32 out of 50 (64%) with 4 (8%), LSIL and 28 (56%), HSIL and 2 (4%) were positive both in urine and cervical scrapes controls. No sample was found positive for HPV type 18.

PCR-based detection revealed that 30 (60%) out of a total of 50 urine samples of pre-cancer lesions were HPV positive. Out of these 50 precancer urine samples, 17 LSIL and 33 HSIL had 4 (8%) and 26 (52%) HPV positives, respectively. HPV infection was detected in the cervical scrape of 35/50 (70%) of precancer lesions with 14% of LSIL and 56% of HSIL found to be HPV positive. However, in control samples (n=50), only 3 (6%) urine samples and 4 (8%) normal cervical samples were found to be HPV positive (Table 1). Subsequently, HPV type specific PCRs performed in these cases to determine the prevalence of two most prevalent HR-HPV types 16 and 18 in urine samples revealed the presence of HPV16 DNA sequence in 28 out of 50 (56%) cervical pre-cancer cases [4(8%) of LSIL and 24 (48%) of HSIL] whereas in cervical scrapes it was 32 out of 50 (64%) with 4 (8%), LSIL and 28 (56%), HSIL and 2 (4%) were positive both in urine and cervical scrapes controls. No sample was found positive for HPV type 18.

**Table 1:** Clinicopathological characteristics and HPV status of cervical pre-cancer, cancer patients and controls.

	Pathology	Number of samples	Total HPV + (HPV L1 Consensus) (n/%)		HPV16 Positive (n/%)		HPV18 Positive (n/%)	
			Urine	Cervical scrape/Biopsy	Urine	Cervical scrape/ Biopsy	Urine	Cervical scrape/Biopsy
Normal /Control		50	3(6%)	4(8%)	2(4%)	2(4%)	0	0
Pre-cancer		50	30 (60%)	35(70%)	28 (56%)	32(64%)	0	0
	LSIL	17	4 (8%)	7(14%)	4(8%)	4(8%)	0	0
	HSIL	33	26 (52%)	28 (56%)	24 (48%)	28(56%)	0	0
Cancer		50	40 (80%)	36* (90%)	37 (74%)	34*(85%)	2 (4%)	2 (4%)
Histopathological grading	WDSCC	28	23(46%)	16(40%)	20 (40%)	14(35%)	0	0
	MDSCC	12	10 (20%)	10(25%)	10 (20%)	10(25%)	0	0
	PDSCC	10	7 (14%)	10 (25%)	7 (14%)	10(25%)	0	0
Clinical stage		50	45 (90%)	46 (92%)	40 (80%)	42 (84%)	0	0
	Stage I	10	10 (20%)	8 (16%)	8 (16%)	8 (16%)	0	0
	Stage II	17	17 (34%)	15 (30%)	14 (28%)	15 (30%)	0	0
	Stage III	18	15(30%)	18 (36%)	15 (30%)	15 (30%)	1 (2%)	1 (2%)
	Stage IV	5	3 (6%)	5 (10%)	3 (6%)	4 (8%)	1 (2%)	1 (2%)

*HPV detection conducted in 40 tissue biopsies.

*HPV* human papillomavirus, *SCC* squamous cell carcinoma, *LSIL* low grade squamous intraepithelial lesions, *HSIL* high grade squamous intraepithelial lesions, *WDSCC* well differentiated SCC, *MDSCC* moderately differentiated SCC, *PDSCC* poorly differentiated SCC.

In cancer cases (n = 50), HPV positivity was detected in 40 out of 50 (80%) urine samples and 36 out of 40 (90%) tumor tissue biopsies. HR-HPV type 16 was the most frequently detected HPV type in the urine (74%) and tumor biopsies (85%) of cervical cancer patients. Interestingly, HPV18 infection was found only in 2 (4%) each in urine and tumor biopsy of cancer patients. We also collected paired adjacent normal tissues as controls from 30 cervical cancer cases which revealed the presence of HPV16 infection in only 4 (8%) cases. Interestingly, none of the paired adjacent normal tissues were positive for HPV 18 infection (Table 1).

When we examined the distribution of HPV prevalence with respect to differentiation and histopathological grades, we obtained HPV positivity in 23 (46%), 10 (20%), 7 (14%) in urine, while 16 (40%), 10 (25%), 10 (25%) in tumor tissue of WDSCC, MDSCC and PDSCC, respectively (Table 1). The HPV status along with histopathological grades and differentiation status of the tumors are presented in Table 1. HPV positivity in urine, cervical scrapes, and tissue biopsies was also correlated with clinical stage of the tumors (Table 1). HPV was found present in urine of 10 (20%) cases of stage I, 17 (34%) of stage II, 15 (30%) of stage III and 3 (6%) in stage IV. Majority of HPV positive tumors had HPV16 genotype regardless of tumor grade and sample types (Table 1). The urine samples of all stage I and II cervical cancer patients were HPV positive, while in paired tissue biopsies it was found that 8 out of 10 cases of stage I and 15 out of 17 of stage II patients were HPV positive. Further, in the urine samples, 15 out of 18 of stage III and 3 out of 5 of stage IV cervical cancer patients were found to be HPV positive, while in paired tumor tissue biopsies, all stage III and stage IV patients were HPV positive (Table 1).

When the results of three paired clinical/biological samples viz. urine, cervical scrapes and tumor biopsies were compared for the presence of HPV infection, there was no significant difference, rather very good correlation of results.

### Expression profiling of selected six miRNAs in paired urine, serum, cervical scrape, and tissue biopsy in control, cervical pre-cancer and cancer

**Figure 1:**
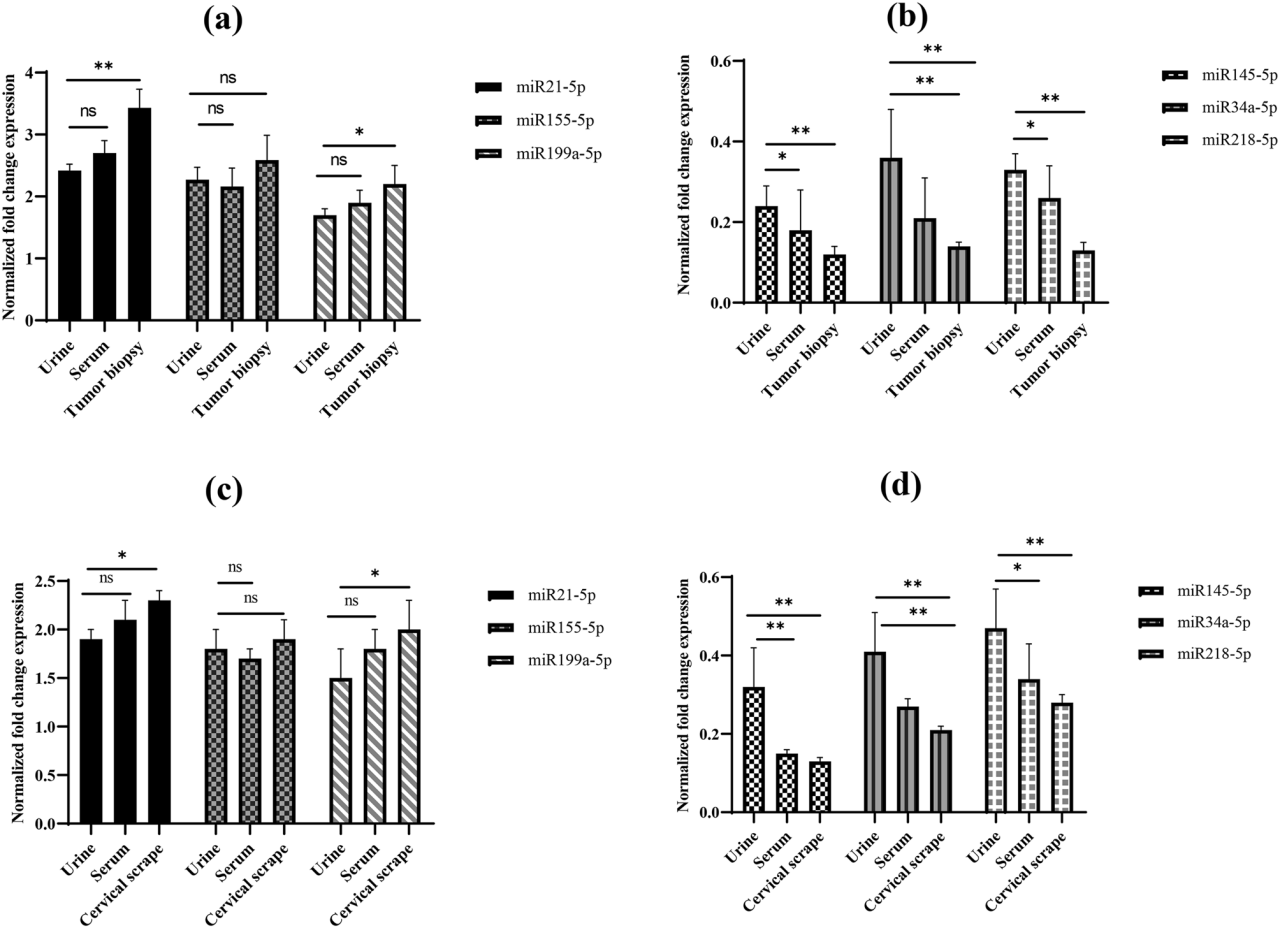
Comparative differential expression profile of six miRNAs in urine, serum, tissue biopsies and cervical scrape derived from healthy controls, cancer and pre-cancer. (**a** & **c**) The miRNA expression level of upregulated miRNAs (miR-21-5p, miR-155-5p, miR-199a-5p) and (**b** & **d**) down regulated miRNAs (miR-145-5p, miR218-5p, and miR-34a-5p). Urine and serum samples were taken from pre-cancer and cervical cancer patients and compared to samples from healthy controls. In case of tissue biopsies, the samples are derived from cancer patients compared with samples from adjacent non-malignant normal tissues. **P ≤ 0.01, ns (nonsignificant), while in case of cervical scrape, the samples are derived from pre-cancer patients and compared with samples from healthy volunteers.

We analyzed the expression of six miRNAs (miR-21-5p, miR-199a-5p, miR-155-5p, miR-145-5p, miR-34a-5p and miR-218-5p) in aforementioned samples using qRT-PCR (Table 2). A total of 460 samples were analyzed for miRNA expression (in 150 urine and serum samples collected from 50 subjects each of normal, cervical pre-cancer, and cancer, 50 cervical scrapes from pre-cancer patient, 40 cervical scrapes from normal controls, 40 tumor tissue biopsies and 30 paired adjacent normal tissues from cervical cancer patients). We found a significant upregulation of miR-21-5p, miR-199a-5p, and miR-155-5p and downregulation of miR-145-5p, miR-34a-5p, and miR-218-5p in urine, paired serum, tumor biopsies and cervical scrape (Table 2, Fig. 1 and Figure S1a, S1b, S1c and S1d) as well as in cervical cancer cell lines (Table S1). Compared to paired adjacent normal tissues, the expression of miR-21-5p (FC = 3.4), miR-199a-5p (FC = 2.2), and miR-155-5p (FC = 2.6) was upregulated while that of miR-145-5p (FC = 0.12), miR-34a-5p (FC = 0.14), and miR-218-5p (FC = 0.13) was downregulated in tissue biopsies of cervical cancer patients (Table 2; Fig. 1a,b; Figure S1c). Similar results were also obtained for cervical scrapes from cervical pre-cancer cases when compared with cytological normal samples (Table 2; Fig. 1c,d, and Figure S1d).

**Table 2:** Relative expression level of miRNAs in paired urine, serum, cervical scrapes, and tissue biopsies.

Sample	Pathology	miR-21-5p (FC ± SE)	*P value*	miR-145-5p (FC ± SE)	*P value*	miR-218-5p (FC ± SE)	*P value*	miR-34a-5p (FC ± SE)	*P value*	miR-155-5p (FC ± SE)	*P value*	miR-199a-5p (FC ± SE)	*P value*
Urine (n = 150) ^a^	Pre-cancer (n = 50)	1.93 ± 0.9	< 0.0001	0.32 ± 0.1	< 0.0002	0.43 ± 0.1	< 0.0003	0.41 ± 0.1	< 0.0001	1.89 ± 0.2	< 0.0001	1.51 ± 0.3	< 0.0001
	Cancer (n = 50)	2.42 ± 0.1	< 0.0001	0.24 ± 0.5	< 0.0002	0.32 ± 0.4	< 0.0001	0.36 ± 0.2	< 0.0001	2.27 ± 0.20	< 0.0001	1.73 ± 0.1	< 0.0001
Serum (n = 150) ^a^	Pre-cancer (n = 50)	2.10 ± 0.20	< 0.0001	0.15 ± 0.01	< 0.0001	0.34 ± 0.09	< 0.0001	0.27 ± 0.2	< 0.0001	1.72 ± 0.1	< 0.0001	1.81 ± 0.2	< 0.0001
	Cancer (n = 50)	2.72 ± 0.2	< 0.012	0.18 ± 0.01	< 0.0001	0.26 ± 0.03	< 0.008	0.21 ± 0.10	< 0.0001	2.16 ± 0.3	< 0.0001	1.92 ± 0.2	< 0.0001
Scrape (n = 90) ^b^	Pre-cancer (n = 50)	2.3 ± 0.1	< 0.0001	0.13 ± 0.1	< 0.0001	0.28 ± 0.02	< 0.0001	0.23 ± 0.01	< 0.0001	1.94 ± 0.2	< 0.0001	2.02 ± 0.3	< 0.0001
Tissue biopsy (n = 70) ^c^	Cancer (n = 40)	3.43 ± 0.3	< 0.0001	0.12 ± 0.2	< 0.0001	0.13 ± 0.2	< 0.0001	0.14 ± 0.2	< 0.0001	2.6 ± 0.4	< 0.0001	2.2 ± 0.3	< 0.0001

*FC* fold change, *SE* standard error.

^a^Including 50 urine and 50 serum samples from normal controls.

^b^Including 40 cervical scrape from normal controls.

^c^Including 30 paired adjacent non-malignant normal tissue.

To determine whether there is a similar trend of miRNAs expression in urine as in tumor biopsies, the correlation of all six miRNAs expression in urine and paired tumor biopsies was calculated using Pearson’s correlation coefficient. Interestingly, the expression of all six miRNAs significantly and positively correlated in urine and paired tumor biopsies of cervical cancer cases (Figure S2) suggesting that miRNA expression profile of urine might accurately reflects to that in tumor cells.

### Correlation of miRNA expression with HPV infection status in HPV + ve, HPV -ve cervical cancer cell lines and samples.

**Figure 2:**
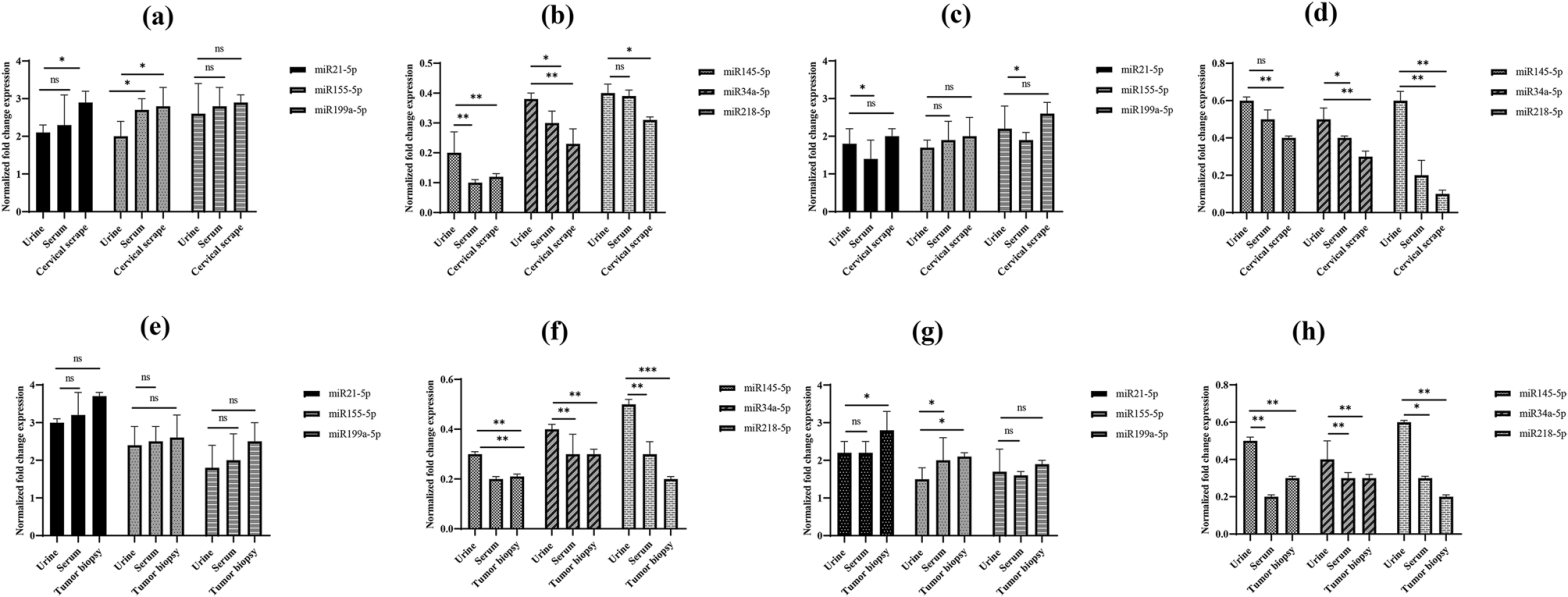
Expression levels of miR-21-5p, miR-155-5p, miR-199a-5p, miR-145-5p, miR-218-5p, and miR-34a-5p in HPV16-positive (**a** & **c**) and HPV16-negative (**b** & **d**) urine, serum, and cervical scrape samples of cervical pre-cancer and in HPV16-positive (**e** & **g**) and HPV16-negative (**f** & **h**) urine, serum, and tissue biopsies of cervical cancer patients.

We checked the expression of all six miRNAs in HPV16 positive cervical cancer cell line SiHa, HPV18 positive cervical cancer cell line HeLa, and HPV negative cervical carcinoma cell line C-33A. Similar to the results in paired urine, serum, cervical scrape and tissue biopsies, qRT-PCR assay showed a marked upregulation in the expression of miR-21-5p, miR-155-5p, and miR-199a-5p and downregulation in the expression of miR-34a-5p, miR-218-5p, and miR-145-5p in HPV positive and negative cell lines (Table S1). The results of expression of upregulated miRNA in paired samples of urine, serum, cervical scrapes and tumor biopsies of HPV positive and negative infection showed no significant difference rather perfect correlation of results with cell lines (Fig. 2).

When we compared the miRNA expression in HPV16 positive and negative samples of cervical pre-cancer and cervical cancer patients, we found that the expression of miR-145-5p was significantly lower in HPV16 positive urine samples of cervical pre-cancer (Figure S3a). Further, the expression of miR-21-5p was significantly higher in the serum of HPV16 positive than HPV negative cervical pre-cancer patients (Figure S3b). Moreover, the expression of miR-21-5p, miR-155-5p, and miR-199a-5p was significantly higher in HPV16 positive than HPV16 negative cervical scrapes of cervical pre-cancer patients (Table S2; Figure S3c ) and the expression of miR-145-5p was significantly lower in HPV16 positive in urine and serum samples of cervical cancer patients (Figure S3d and S3e) and the expression of miR-21-5p, miR-155-5p, and miR-199a-5p was significantly higher in HPV16 positive than HPV16 negative tissue biopsies of cervical cancer patients (Figure S3f.).

### Correlations of miRNA expression level with clinicopathological characteristics of cervical pre-cancer and cancer.

The expression of all six miRNAs in urine, serum, cervical scrape, and tissue biopsies was correlated with various clinicopathological characteristics and known risk factors for cervical cancer. Except miR-34a-5p in the urine and miR-34a-5p and miR-199a-5p in the serum, the expression of all other miRNAs was significantly correlated with the age of the cervical pre-cancer patients (Table S3). The expression of miR-21-5p (in urine and serum), miR-145-5p (in urine), miR-155-5p (in serum), and miR-199a-5p (in urine and serum) correlated with the age of marriage of cervical pre-cancer patients (Table S3). Further, the expression of miR-21-5p (in urine and serum), miR-145-5p (in serum), and miR-199a-5p (in urine and serum) correlated with the parity of cervical pre-cancer patients (Table S3).

In cervical cancer patients, the expression of miR-21-5p (in serum), miR-218-5p (in urine and serum), miR-155-5p (in urine and serum), and miR-199a-5p (in serum) were correlated with the age (Table S3). Further, the expression of miR-21-5p, miR-155-5p, and miR-199a-5p in urine and serum and the expression of miR-145-5p and miR-218-5p in serum showed correlation with the age of marriage of cervical cancer patients (Table S3). The expression of all 6 miRNAs in urine and serum significantly correlated with the stage of cervical cancer (Table S3).

In cervical scrapes of pre-cancer patients, we found a significant correlation between the expression of miR-145-5p, miR-218-5p, miR-34a-5p, and miR-199a-5p with age (Table S3). Further, the expression of miR-21-5p and miR-155-5p correlated with the age of marriage while that of miR-21-5p, miR-155-5p, and miR-199a-5p significantly correlated with parity (Table S3). In tissue biopsies of cervical cancer patients, except miR-145-5p and miR-34a-5p, the expression of all other miRNAs significantly correlated with the age (Table S4). Additionally, the expression of all 6 miRNAs in tumor biopsies significantly correlated with the stage of cervical cancer patients (Table S4).

### Diagnostic performance of miRNAs for cervical cancer as revealed by ROC analysis

**Figure 3:**
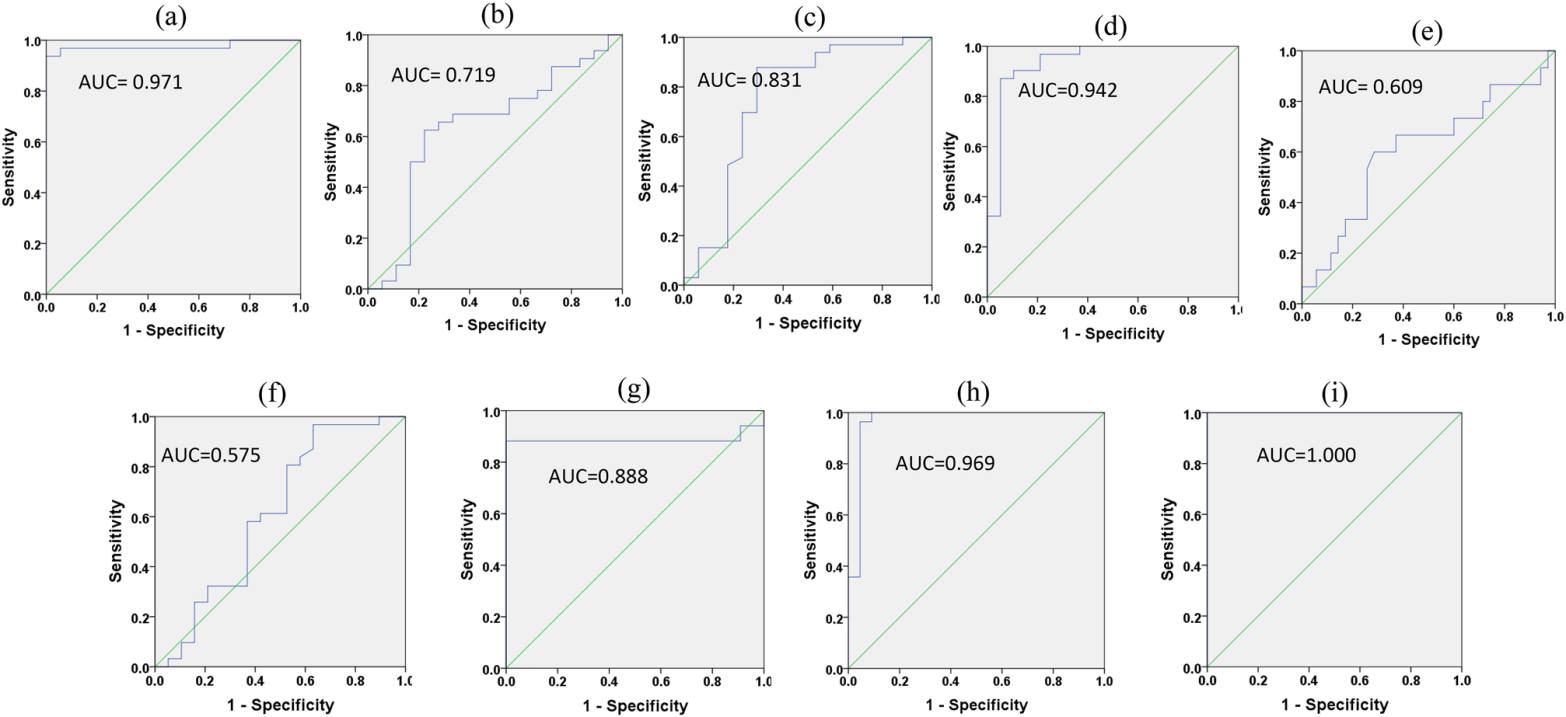
Receiver operating characteristics (ROC) plots of miRNA expression in urine sample of cervical cancer patients. ROC plots evaluating diagnostic potential of (**a**) miR-21-5p, (**b**) miR-155-5p, (**c**) miR-199a-5p, (**d**) combined ROC plots of three upregulated miRNAs (miR-21-5p, miR-155-5p, and miR-199a-5p), (**e**) miR-218-5p, (**f**) miR-145-5p, (**g**) miR-34a-5p, (**h**) combined ROC plots of three downregulated miRNAs (miR-218-5p, miR34a-5p, and miR-145-5p), and (l) combined ROC plots of all six miRNAs.

To evaluate the diagnostic utility of urinary and tissue miRNAs for cervical cancer, ROC analysis was performed (Table 3; Fig. 3 and S4). ROC analysis demonstrated that urinary miRNAs may serve as useful non-invasive biomarkers for discriminating cervical pre-cancer and cancer from healthy controls. miR-21-5p exhibited the best AUC (0.971) with a sensitivity and specificity of 88% and 98%, respectively (Table 3). In an attempt to improve the diagnostic performance of miRNAs, we constructed a binary logistic regression model to evaluate the performance of the combined use of 3 or all 6 urinary miRNAs together. Interestingly, a combination of miR-145-5p, miR-218-5p and miR-34a-5p yielded a 100% sensitivity and 92.8% specificity for distinguishing cervical cancer patients from healthy controls (Table 3). As evident from Table 3, the diagnostic performance of miRNA expression evaluated in tumor biopsies was better than urinary miRNAs. A combination of 3 miRNAs (miR-21-5p, miR-155-5p, and miR-199-5p) as well as all 6 miRNAs yielded a 100% sensitivity and specificity for distinguishing cervical cancer patients from healthy controls (Table 3). The absolute sensitivities and specificities achieved after combining multiple miRNAs indicate that detection of a combination of miRNAs in liquid biopsy sample like urine can prove to be a useful biomarker for the early diagnosis of cervical pre-malignant lesions and for reducing the incidence of cervical cancer and better management.

**Table 3:** Diagnostic performance of miRNAs in cervical cancer patients.

miRNAs	Source	Sensitivity (%)	Specificity (%)	AUC (95%CI)	Cut-off	*P value*
miR-21-5p	urinary miRNA	88	98	0.971(0.957–0.985)	1.912	< 0.0001
miR-155-5p	urinary miRNA	86.7	91.7	0.719(0.816–0.907)	0.8312	< 0.0001
miR-199a-5p	urinary miRNA	93.8	88.3	0.831(0.869–0.945)	0.8964	< 0.0001
miR-145-5p	urinary miRNA	89.1	91.7	0.575(0.743–0.407)	0.2387	< 0.0001
miR-218-5p	urinary miRNA	67.7	60	0.609(0.729–0.482)	0.4983	< 0.0001
miR-34a-5p	urinary miRNA	83.3	72.7	0.888 (0.781–1.00)	0.0186	< 0.0001
Combined three downregulated miRNA (miR-21-5p, miR-155-5p, miR-199-5p) signature	urinary miRNAs	100	78.9	0.942 (0.868–1.00)	0.5104	< 0.0001
Combined three upregulated miRNA (miR-145-5p, miR-218-5p,miR-34a-5p) signature	urinary miRNAs	100	92.8	0.969 (0.912–1.000)	0.7877	< 0.0001
Combined ROC of all six miRNAs	urinary miRNAs	100	100	1.000(1.00–1.00)	− 1.000	< 0.0001
miR-21-5p	Tumor biopsy miRNA	98	85	0.993 (0.975–1.000)	1.182	< 0.0001
miR-155-5p	Tumor biopsy miRNA	95	72.9	0.976 (0.935–1.000)	0.7562	< 0.0001
miR-199a-5p	Tumor biopsy miRNA	96.5	92.7	0.978(0.964–1.000)	1.302	< 0.0001
miR-145-5p	Tumor biopsy miRNA	90	75	0.946 (0.881–1.000)	0.1546	< 0.0001
miR-218-5p	Tumor biopsy miRNA	85	92.7	0.995 (0.983–1.000)	0.0652	< 0.0001
miR-34a-5p	Tumor biopsy miRNA	73.2	91	0.826 (0.713–0.940)	0.2269	< 0.0001
Combined three downregulated miRNA (miR-21-5p, miR-155-5p, miR-199-5p) signature	Tumor biopsy miRNAs	100	100	1.000(1.000–1.000)	− 1.000	< 0.0001
Combined three upregulated miRNA (miR-145-5p, miR-218-5p, miR-34a-5p) signature	Tumor biopsy miRNAs	100	88.9	0.932 (0.839–1.000)	0.4202	< 0.0001
Combined ROC of all six miRNAs	Tumor biopsy miRNAs	100	100	1.000(1.000–1.000)	− 1.000	< 0.0001

### Prognostic value of miRNA expression levels in cervical cancer

We constructed a prognostic signature by integrating the expression profiles of six miRNAs after a median follow up of 12–15 months and calculated corresponding estimated regression coefficient. To evaluate the ability of these miRNAs as a signature to predict prognosis, we first carried out a univariate Cox proportional hazards regression analysis to evaluate the correlation between each of the six miRNAs and the survival outcome. The analysis revealed a significant correlation of miR-155-5p, miR-21-5p, miR-199-5p, miR-218-5p, and miR-34a-5p expression with the overall survival (OS) (Table 4). We also performed multivariate Cox regression analysis to find independent prognostic power of aforementioned miRNAs within the context of several common clinical parameters, including age at diagnosis, age of marriage, parity, HPV infection, clinical stage and histopathological grade. The multivariate analysis revealed that miR-34a-5p (HR = 5.721; *P* = 0.003), miR-218-5p (HR = 0.218; *P* = 0.011), and HPV 16 (HR = 0.352; *P* = 0.08) were independent prognostic factors of OS (Table 4). Further, a combination of 3 upregulated miRNA signature (HR = 3.584; *P* = 0.01) and 3 downregulated miRNA signatures (HR = 0.239; *P* = 0.01) also retained their significance as independent prognostic factors of OS while performing univariate analysis, however, it was not significant in multivariate analysis (Table 4).

**Table 4:** Univariate and multivariate Cox regression analysis to evaluate the prognostic value of individual miRNAs.

miRNA	Univariate analysis		Multivariate analysis*	
	HR (95% CI)	*P value*	HR (95% CI)	*P value*
miR-155-5p	0.148 (0.051–0.431)	< 0.001	1.081 (0.384–3.046)	0.883
miR-21-5p	0.313 (0.129–0.759)	0.01	0.843 (0.197–3.602)	0.801
miR-199-5p	0.132 (0.032–0.557)	0.006	2.769 (0.696–11.023)	0.14
miR-218-5p	4.175 (1.34 -13.0)	0.01	0.218 (0.068–0.702)	0.011
miR-34a-5p	6.248 (1.44–26.96)	0.01	5.721 (1.786–18.325)	0.003
miR-145-5p	1.776 (0.891–3.518)	0.103	3.996 (0.880–18.140)	0.07
Age of marriage	1.580 (0.702–4.516)	0.219	1.333 (0.426–4.176)	0.622
HPV 16	0.225 (0.103–0.495)	< 0.001	1.352 (0.110–3.132)	0.08
Age	0.968 (0.917–6.031)	0.949	0.135 (0.026–0.698)	0.017
Parity	2.351 (0.095–1.308)	0.075	0.594 (0.251–1.408)	0.237
Histopathological grade	1.159 (0.367–3.665)	0.802	0.947 (0.192–4.677)	0.947
Three-up regulated miRNA signatures (high risk vs. low risk)	3.584 (1.23–10.410)	0.01	0.833 (0.191–3.637)	0.809
Three downregulated miRNA signatures (high risk vs. low risk)	0.239 (0.072–0.787)	0.01	0.445 (0.113–1.745)	0.245
Clinical stage (I–II)	1.826(0.865–3.853)	0.114	0.248 (0 .091–0.672)	0.006
Clinical stage (III–IV)	0.337 (0.133–0.852)	0.02	1.996 (0.144–27.598)	0.606

*HR* hazard ratio, *CI* Confidence interval.

*Adjusted with age at diagnosis, age of marriage, parity, HPV infection, and tumor stage and grade.

**Figure 4:**
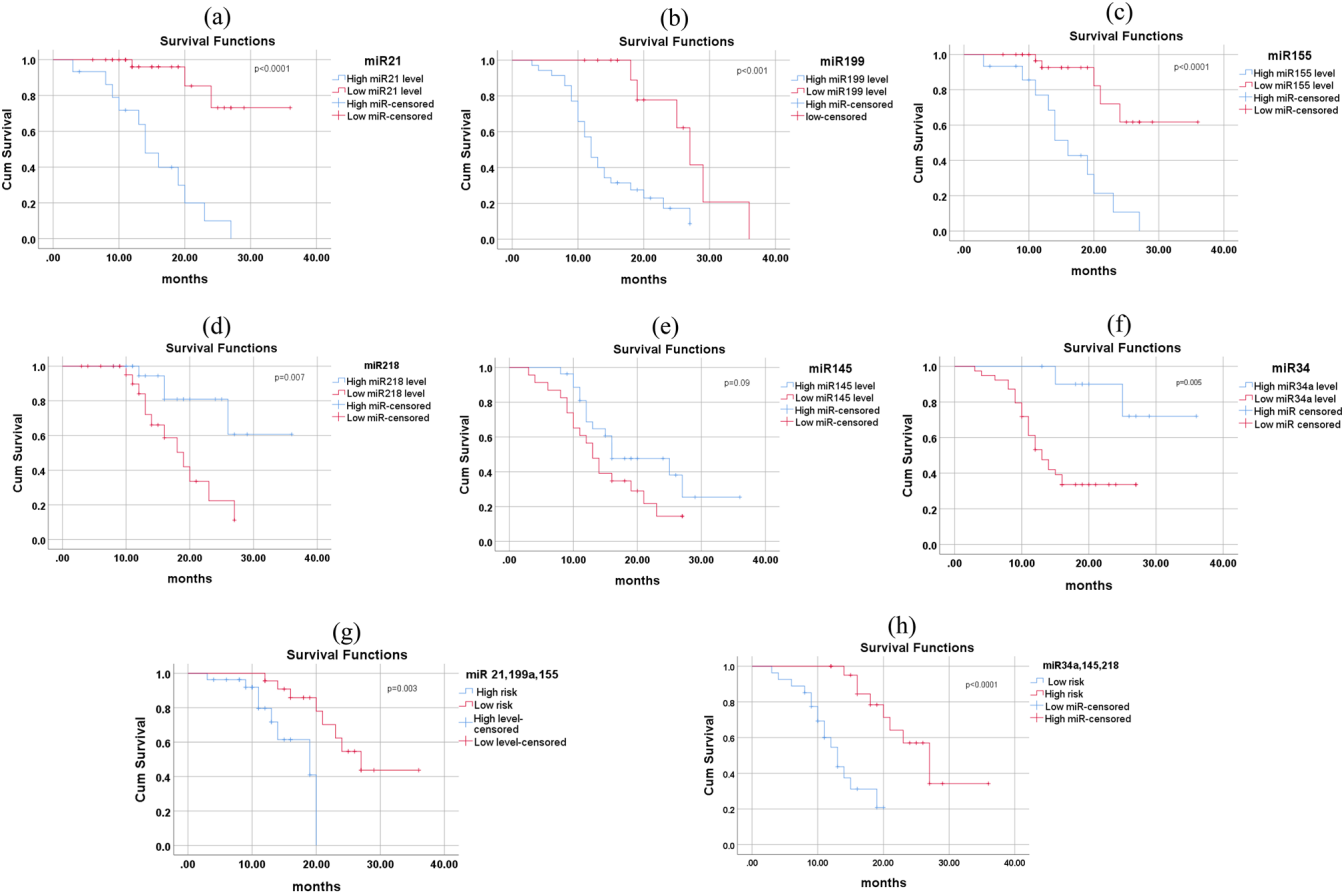
The correlation of miRNA expression with overall survival of cervical cancer patients calculated using Kaplan Meier curve and Log-rank test. The patients were stratified into high expression and low expression group according to median of each miRNA. (**a**) miR-21-5p, (**b**) miR-199a-5p, (**c**) miR-155-5p, (**d**) miR-218-5p, (**e**) miR-145-5p, (**f**) miR-34a-5p, (**g**) three upregulated miRNA signatures, and (**h**) three downregulated miRNA signatures. The patients were stratified into high-risk group and low risk group based on median.

Further, we have done the Kaplan Meier survival analysis to examine the potential prognostic power of the six miRNAs (Fig. 4). Notably, different expression levels of miR-155-5p, miR-21-5p, miR-199a-5p, miR-218-5p, miR-34a-5p have led to statistically significant different survival rates (log-rank test *P*-value < 0.0001, *P* < 0.001, *P* < 0.0001, *P* = 0.007 and *P* = 0.006, respectively), while no survival differences were observed between the high and low expression set of miR-145-5p (*P*-value = 0.09). We divided these six miRNAs among all samples into a high expression group and a low expression group to the cut off expression level.

### Target gene prediction and functional analysis of miRNAs

To further understand the functions of all 6 miRNAs, the online target gene prediction was performed using three prediction algorithms: miRDB, Target Scan, and microT-CDS. As evident from Figure S5, all 6 miRNAs can potentially regulate the expression of a large number of genes. The number of overlapping targets for miR-21-5p, miR-155-5p, miR-199a-5p, miR-34a-5p, miR-145-5p, and miR-218-5p were 274, 208, 234, 568, 1708, and 508, respectively (Figure S5). Subsequently, pathway enrichment analysis was performed on all overlapping potential target genes of six miRNAs to investigate their potential biological functions (Figure S6A). We observed that these genes were involved in PI3K-AKT pathway, mTOR signaling pathways, microRNA in cancer pathway along with other pathways involved in cancer (Figure S6B) as per the analysis performed using DAVID^25,56^. The pathway analysis revealed the enrichment of genes involved in signaling transduction, RNA polymerase II transcription pathway, and immune system. Since most of these pathways are well known for their role in tumor cell proliferation, angiogenesis, apoptosis, tumor cell migration and invasion, we can emphasize that these 6 miRNAs can be involved in the development, progression and prognosis of cervical cancer.

## Discussion

HPV infection is well recognized as the major risk factors for cervical cancer which may take 10–20 years after getting HPV infection to develop invasive cervical cancer. Hence, cervical cancer represents a suitable model not only to dissect molecular determinants of cancer initiation and progression but also to identify cancer biomarkers for early diagnosis and prognosis of cervical cancer. Identification of cancer biomarker using non-invasive methods is highly useful for easy screening, early diagnosis, monitoring treatment response, risk assessment and cancer control. Hence, we explored the clinical utility of urinary miRNAs as non-invasive diagnostic and prognostic biomarkers in cervical pre-cancer and cervical cancer patients. Urine generally contains a mixture of circulating cells and local cells from epithelial wall of the cervix, vaginal discharge, menstrual blood, and cervical exfoliated cells containing HPV and miRNAs in the urine might be secreted or shaded from any of the origins mentioned above. To avoid the mixing of urothelial or other cells was minimised by collecting the midstream urine by excluding 5–7 ml of first void urine. We observed a significant positive correlation of miRNA expression in urine with paired serum, cervical scrapes or tumor tissue of cervical cancer patients indicating that the analysis of miRNA or any genetic or epigenetic alterations in urine cells may offer a better and reliable alternative biological material to tissue biopsy for an easy diagnosis and prognosis of cervical cancer.

In the present study, we evaluated the expression of 6 important miRNAs (miR-21-5p, miR-199a-5p, miR155-5p, miR-34a-5p, miR-145-5p, and miR-218-5p) selected based on results of several previous studies complete database search^19,20^. Some of these miRNAs are also found to be associated with HR-HPV infection in cervical pre-cancer and cancer^21,22^. The expression level of six urinary miRNAs was found to be significantly altered (Upregulated: miR-21-5p, miR-199a-5p, and miR-155-5p; Downregulated: miR-145-5p, miR-34a-5p, and miR-218-5p) in our cohort of cervical pre-cancer and cancer patients as compared to that of healthy controls. The expression level of 6 selected miRNAs in urine exhibited similar trends as in paired tissue biopsies, cervical scrapes and serum of precancer and cancer patients including cervical cancer cell lines. The fold-change in the expression level of miR-21, miR-155 and miR-199a was higher in cervical scrapes than in serum and urine of precancer cases; but certainly, significantly higher than controls. On the contrary, the fold-change in the expression level of miR-145, miR-34a and miR-218 was higher in urine than in serum and cervical scrapes and the difference in fold-change among various sample types was found to be statistically significant. The upregulation of urinary miR-21, miR-155, and miR-199a among precancer and cancer cases align with earlier studies which demonstrated overexpression of these miRNAs in cervical scrape and tumor biopsy in cervical cancer by invasive method^23–25^. The correlation between these oncomiRs expression in paired urine, serum, cervical scrapes and tumor biopsy and cervical cancer cell lines suggests that urinary miRNAs could be used as a potential biomarker for investigating cervical abnormalities. The downregulation of urinary miR-145, miR-34a and miR-218 in cervical precancer and cancer when compared with normal healthy control is in line with an earlier study which showed that downregulation of these three miRNAs in the cervical scrape and tumor tissue correlate well with malignant transformation^26^.

To evaluate the diagnostic value of urinary miRNAs in cervical cancer, ROC analysis was performed for individual miRNA^27,28^. Among the six miRNAs studied, miR-21-5p was found to be the best diagnostic biomarker (AUC = 0.971) for differentiating the cervical cancer patients with the 88% sensitivity and 98% specificity. Further, ROC analysis of a combination of all 6 urinary miRNAs revealed absolute sensitivity and specificity of miRNA signature in discriminating cervical pre-cancer and cancer patients from normal individuals. Such high sensitivity and specificity of the urinary miRNA signature indicates that they could serve as a potential biomarker for the early diagnosis of cervical pre-malignant lesions and cancer. If urinary miRNA of pre cancer with distinctly differential ROC value particularly for low grade squamous intraepithelial lesion (LSIL), can be detected early and treated effectively, leading to the reduction of cervical cancer incidence.

We also analyzed the clinical utility of miRNAs as prognostic biomarkers by correlating their expression with overall survival (OS) of cervical cancer patients. We found that miR-34a-5p and miR-218-5p are significantly associated with overall survival of cervical cancer patients in a multivariate analysis indicating their utility as independent prognostic biomarkers. A combination of 3 upregulated miRNA signature and 3 downregulated miRNA signature appears to be independent prognostic factors of OS suggesting that multi-miRNA-based model may provide more powerful information for predicting the prognosis of cervical cancer patients, though not significant in multivariate analysis which may be due to low sample size.

Previous studies have demonstrated that these miRNAs are crucial for the initiation, progression, and metastasis of cervical cancer by regulating various signaling pathways, including cancer cell proliferation, differentiation, apoptosis, adhesion, cell cycle arrest, migration, and invasion^29,31^. miR-21 has been shown to be highly elevated in cervical cancer^32,36^ and it promoted cell proliferation and invasion by targeting programmed cell death protein 4 (PDCD4) in cervical cancer^33^, while miR-155 did the same by targeting SOCS1, C/EBPβ, and SHIP1^24,34–36^. Lee et al. (2010) demonstrated upregulation of miR-199a in cervical cancer and is considered as a potential therapeutic target for cervical cancer^25,36,37^.The expression of miR-218, which can target *LAMB3*^26^, was downregulated in cervical cancer following interaction with HR-HPV infection. The expression of miR-145 was also found to be downregulated in cervical cancer tissues and cell lines and was associated with tumor size, lymph node metastasis, HPV16 infection, and poor prognosis^38–41^. Further, miR-34a has been reported to be downregulated in cervical cancer cell lines HeLa, SiHa, C4I, C33a, and CaSki affecting Notch and Jagged1 proteins^42^. Together these findings indicate that these six miRNAs may serve as potential target(s) for effective diagnosis, therapy and prognosis of cervical cancer.

We also examined the significance of HPV infection as such, as it is the principal etiological agent responsible for transformation of normal cervical epithelium to pre-neoplastic cervical intraepithelial neoplasia (CIN) which subsequently undergoes malignant transformation leading to development of invasive cervical cancer. However, the exact mechanism is yet unclear. Proteins encoded by E6 and E7 genes of HR- HPVs bind to and degrade p53 and Rb proteins respectively^38,43^. These six HPV-associated miRNAs that are directly or indirectly regulated by HPV E5-E7 proteins, play an important role in the initiation and progression of cervical cancer^21,40^. The expression of miR-21-5p, miR-199-5p, and miR-155-5p was found to be significantly increased in HPV16 positive than HPV16 negative cervical cancer patients. These results suggest that the aberrant expression of these miRNAs might be the consequence of HR-HPV induced cervical carcinogenesis^23,36,44^. During virus induced cellular transformation, HPV E6 and E7 as oncoproteins also interact with many other signaling pathways, including the miRNA pathways^38,39,43,44^. The expression of miR-199a-5p, miR-218-5p, miR-145-5p, and miR-34a-5p was also reported to be regulated by HPV^20,27,39^. The upregulation of miR-21 in cervical cancer was observed in association with the dysregulation of DROSHA, an important enzyme in the miRNA biogenesis pathway, and it is suggested to be modulated by HPV16 E6/E7^21,32^. HPV E6/E7 was also found to suppress miR-145-5p expression^41^.

Furthermore, we screened the target genes of the six miRNAs and predicted the enrichment pathways and biological functions of target genes using bioinformatic tools as miRNAs have been considered as the master modulators of multiple biological and pathological pathways during cancer development. To gain a deeper insight into the molecular functions of these miRNAs, we predicted the target genes and analyzed the related pathways and GO annotations. We used databases and 3 different online miRNA target prediction tools and used only those targets which were predicted by all 3 programs. We observed that these six miRNAs could regulate several key signaling pathways, including MAP kinase pathways, AMP kinase pathways, focal adhesion, cGMP-PKG, Wnt/ β-catenin, and mTOR signaling pathways. Accumulating evidence suggests that activation of MAP kinase pathway is important in cervical cancer progression, invasion, and metastasis^45,46^. Yung *et. al*. (2013) and Kwan *et. al.* (2013) reported that activation of AMP-activated protein kinase (AMPK), a metabolic sensor, hinders cervical cancer cell growth through reducing the expression of FOXM1 and blocking the DVL3 mediated Wnt/β-catenin signaling pathway^45,46^. Also, transformation of human keratinocytes with HPV oncoproteins E6 and E7 requires activation of the Wnt/β-catenin pathways and β-catenin relocate from cytoplasm to nucleus as the cervical cancer progresses. This activation may serve as a screening tool in HPV-infected population to detect malignant progression and prognosis^43^. Additionally, it has been well recognized that the PI3 kinase/Akt/mTOR signaling pathway plays a pivotal role in development of cervical cancer and inhibition of mTOR kinase activity suppress tumor development^45^. Therefore, further studies in larger sample size are required to validate these observations as it can provide new avenues for developing effective therapeutic interventions in cervical cancer.

Although, this is the first study to select miRNA expression profile in urine to demonstrate that these miRNAs can reliably be used not only for early diagnosis, but also can serve as prognostic biomarkers for cervical pre-cancer and cancer patients, we feel that there remain certain limitations. One of the limitations is that we could not control all variables which could influence miRNA expression profile in the urine samples. These include use of diuretic drugs, alcohol intake, water intake, temperature stability of miRNAs in the urine and storage time. However, some studies have shown that urinary miRNA is relatively stable under various storage conditions^47^. Also, almost all women in our study were not alcohol users. Another limitation is the use of snRNA U6 for normalizing the expression of target miRNAs. Normalization of miRNA being the most controversial, there is no universal endogenous control and use of different controls could yield different results. Also, there could be contamination of epithelial cells in the urine from urinary system. Regardless of the above-mentioned limitations and it could be further validated in a larger patient cohort, yet the study demonstrates the potential utility of urinary miRNAs as reliable biomarkers for non-invasive screening, diagnosis, prognosis and therapeutics target(s) of cervical cancer.

## Conclusion

A set of six specific urinary miRNA signatures (miR-21-5p, miR-155-5p, miR-199a-5p, miR-145-5p, miR-218-5p and miR-34a-5p) has been identified and characterized with oncogenic and tumor suppressor potential, and can serve as superior diagnostic and prognostic biomarkers with high sensitivity and specificity for cervical pre-cancer and cancer. However, larger prospective studies comparing these miRNA signatures with cytology and HPV may be carried out to further validate the present findings before the use of urinary miRNAs as reliable non-invasive biomarkers for cervical and other cancers.

## Materials and methods

### Collection and processing of biological samples

For this prospective study, we recruited 150 subjects comprising of 50 subjects each of cervical pre-cancer, cervical cancer, and healthy controls from the outpatient department of Gynecology and Obstetrics, Safdarjung Hospital, New Delhi. The sample size (N = 150) has been calculated using the standard formula N = z^2^ [P(1 − P)/(D^2^)] where, N is required sample size, Z is the confidence level, P is the prevalence and D is the margin of error. The prevalence of HPV infection in cervical cancer and healthy controls was taken as 85% and 11%, respectively. The D value taken as 10% and Z value as 95% for all the cases.

Cervical scrapes from pre-cancer lesions were collected from low or high grade squamous intraepithelial lesion (LSIL/HSIL) patients. Tumor tissue biopsies were collected from patients undergoing surgical resection or radical hysterectomy for invasive cervical cancer. From all patients, urine was collected before the collection of cervical scrapes or biopsies. The study was approved by Institutional Ethics Committee of Amity University, Uttar Pradesh (AUUP), Noida (India) and Institutional Ethics Committee of V.M. Medical College & Safdarjung Hospital, New Delhi (India). Written informed consent was obtained from all subjects. The study was carried out in accordance with the guidelines and principles of the Helsinki Declaration.

Thirty to fifty ml of midstream urine was collected after excluding 5–7 ml of first void urine to exclude mixing of urothelial and other cells and stored at 4 °C for ~ 2hours before processing. Urine sample from each patient was divided into two parts and centrifuged at 3,000 rpm for 10 min. The resulting pellet was washed twice with sterile 1 × phosphate buffer saline (PBS pH 7.4). For the detection of HPV, pellet was suspended in 1 ml of 1 × PBS (pH 7.4) and stored at − 20 °C. For RNA isolation, pellet was mixed with 1 ml of TRIzol reagent (Invitrogen, Carlsbad, CA, USA) and stored at − 80 °C. Cervical scrapes obtained from cervical pre-cancer patients and healthy controls, by scraping the ectocervix with a wooden spatula or cytobrush, and tissue biopsies obtained from cervical cancer patients were collected in 1 × PBS (pH 7.4) and TRIzol reagent (Invitrogen, Carlsbad, CA, USA). Serum was prepared from 2 ml of venous blood, which was allowed to clot for 30 min at room temperature followed by centrifugation at 3,000 rpm for 10 min.

### Cell culture

Human cervical cancer cell lines SiHa (HPV16 + ve), HeLa (HPV18 + ve), and C33a (HPV − ve) were obtained from the ATCC. The cells were cultured in Dulbecco’s Modified Eagle Medium (DMEM, Sigma Aldrich) supplemented with 10% heat-inactivated fetal bovine serum (FBS, Sigma Aldrich), 2 mM L-glutamine (Sigma Aldrich) and antibiotics, penicillin (100U/ml) and streptomycin (100 μg/mL) (Sigma Aldrich) at 37 °C with 5% CO_2_ and 95% humidity in CO_2_ incubator.

### HPV detection and genotyping

Genomic DNA was extracted from urine samples, cervical scrapes and tumor biopsies using standard proteinase K digestion, phenol/chloroform extraction and ethanol precipitation as described previously^18,48–50^. The quantity and quality of DNA was assessed using agarose gel electrophoresis and by UV spectrophotometry (Nanodrop, Thermo Fisher Scientific). HPV detection was done by PCR using a pair of HPV L1 consensus primers MY09 and MY11 that gave an amplicon of 450 bp (Table S5). HPV genotyping was done by PCR using type-specific primers for predominant HPV types 16 and 18 (Table S5). PCR was performed in a 25 µl reaction mixture containing 100 ng DNA, 10 mM Tris–HCl (pH8.4), 50 mM KCl, 1.5 mM MgCl_2_,125 mM of each dNTPs (Thermo Fisher Scientific), 5 pmol of oligonucleotide primers and 0.5U Taq DNA polymerase. β-globin gene was used as an internal control. Rest of the method was standard procedures routinely followed in our lab^18^.

### RNA extraction and cDNA synthesis

Total RNA extraction from urine, cervical scrapes, tissue biopsies, and cervical cancer cell lines was done using TRIzol reagent (Invitrogen, Carlsbad, CA, USA) according to the manufacturer’s instruction. Total RNA was isolated from 250 µl serum using TRIzol LS reagent (Invitrogen, Carlsbad, CA, USA) according to manufacturer’s instructions. RNA concentration and purity were determined by Nanodrop (Thermo Fisher Scientific). cDNA synthesis was done from total RNA using High-Capacity cDNA synthesis kit (Thermo Fisher Scientific). Briefly, miRNAs were reverse transcribed using 500 ng of total RNA and pools of miRNA-specific stem loop primers following manufacturer’s instructions (Table S6). The cDNA was stored in − 20 °C and later used for quantitative reverse transcription polymerase chain reaction (qRT-PCR).

### Analysis of miRNA expression using qRT-PCR

qRT-PCR reactions were performed separately for RNA extracted from urine, serum, cervical scrape, tumor tissue biopsies, and cell lines with their corresponding normal controls using SYBR Green (Applied Biosystems, USA). snRNA U6 was used as a reference control. The threshold cycle data were determined using the default threshold settings. Same concentration of cDNA was used for all miRNA analysis in order to maintain consistency and same efficiency. The reaction conditions were 10 min at 95 °C, followed by 45 cycles of 95 °C for 15 s and 60 °C for 1 min. All experiments were run in triplicate, along with a negative and a positive controls. The fold change of miRNA expression for each sample in relation to normal control was calculated based on the threshold cycle (CT) value using the following formula: Relative Quantification (RQ) = 2^−ΔΔCT^^49,51^.

### Target gene prediction and enrichment analysis

The potential target genes of candidate miRNAs were predicted through three different online algorithm miRDB (http://www.mirdb.org/miRDB)^52,53^,TargetScanv7.1 (http://www.targetscan.org/) and DIANA microT-CDS web Server v 5.0 (http://www.microrna.gr/webServer)^54^. To further support the consistency of the bioinformatics analysis, the overlapping target genes from three online tools of each miRNA were identified using a Venn diagram (https://omics.pnl.gov/software/venn-diagram-plotter)^55,56^. The gene ontology enrichment was conducted by The ShinyGO v0.60: Gene Ontology Enrichment Analysis (http://bioinformatics.sdstate.edu/go/)^57^ and pathway enrichment of overlapping target genes was conducted through The Database for Annotation, Visualization and Integrated Discovery (DAVID v6.7) (http://david.nicfcrf.gov)^25,56^ bioinformatics online tool. *P* < 0.05 was set as the cut-off criteria.

### Statistical analysis

Data were analyzed using Graph pad prism statistics software program version 5 and IBM SPSS statistics software program version 26. The data was exhibited as the mean ± SD (standard deviation). Mann–Whitney test as well as student’s t-test was employed to compare the significant mean difference of miRNAs expression correlation between two groups. Kruskal–Wallis test was done to check the significant mean difference between different sampling groups. The correlation analysis to assess the relationship between miRNA expression and clinical features was performed by Pearson's correlation coefficient. The diagnostic performance of miRNAs was checked by plotting receiver operator characteristic (ROC) curve. The prognostic value was assessed by employing Kaplan–Meier curve and Long-rank method for each differentially expressed miRNA. The miRNAs that were significantly associated with OS were identified as prognostic miRNAs and then subjected to a binary logistic regression analysis. Subsequently, a prognostic miRNA signature was constructed for calculating a risk score for each cervical cancer patient. With the miRNA signature, cervical cancer patients were classified into high risk (high expression) and low risk (low expression) groups using the median risk score.

Kaplan–Meier method was employed for evaluating the differences in patients’ survival between low risk and high-risk groups. Cox proportional hazards regression analysis was carried out for evaluating the impact of miRNAs on survival time and clinical survival of the patients. Patients who had poor chances of survival were the ones having high-risk score^56^. We used the Z score from the Cox regression model as the coefficient for each miRNA and established a single prognostic model. The risk score for predicting survival time was calculated by the following formula:

$${\text{Risk}}\;{\text{score}} = {\text{Z}}\;{\text{score}}*{\text{E}}_{{{\text{mir}}1}} + {\text{Z}}\;{\text{score}}*{\text{E}}_{{{\text{mir}}2}} + {\text{Z}}\;{\text{score}}*{\text{E}}_{{{\text{mir}}3}}$$

where E=level of miRNA expression.

## Supplementary Information

Supplementary Information.
