# Supplementary material for: Urine miRNA signature as a potential non-invasive diagnostic and prognostic biomarker in cervical cancer
Source: Sci Rep. 2021 May 14;11:10323. doi: 10.1038/s41598-021-89388-w (PMC8121812; doi:10.1038/s41598-021-89388-w)
Supplement: Supplementary file 1 — Supplementary Information. [file 41598_2021_89388_MOESM1_ESM.docx]

**SUPPLEMENTARY INFORMATION**

**Urine miRNA signature as a potential non-invasive diagnostic and prognostic biomarker in cervical cancer**

Mehreen Aftab^1^, Satish S. Poojary^1^, Vaishnavi Seshan^2^, Sachin Kumar^3^, Pallavi Agarwal^1^, Simran Tandon^1^, Vijay Zutshi^2^, Bhudev C. Das^1*^

^1^Amity Institute of Molecular Medicine & Stem Cell Research (AIMMSCR), Amity University Campus, Sector-125, Noida, 201313, Uttar Pradesh, India

**^2^**Department of Gynaecology and Obstetrics, Safdarjung Hospital, New Delhi-110029, India

**^3^**Deptatment of Medical Oncology, Dr. B R Ambedkar Institute Rotary Cancer Hospital, All India Institute of Medical Sciences, Ansari Nagar, New Delhi-110029, India

*Correspondence and materials request should be addressed to B.C.D (bcdas@amity.edu)


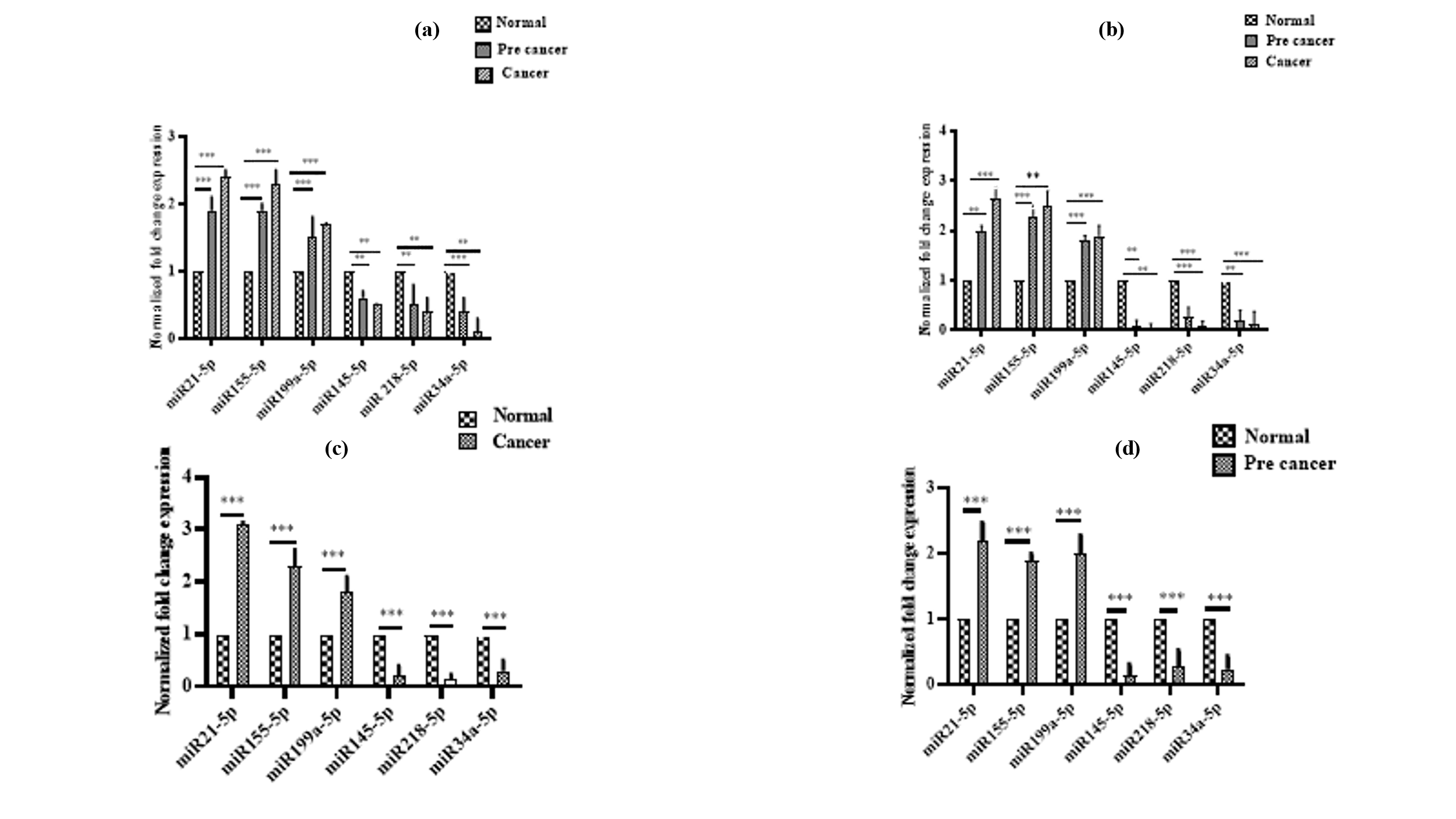
**SUPPLEMENTARY FIGURES**

**Figure S1:** Differential expression of six miRNAs in urine, paired serum, tissue biopsies and cervical scrape derived from healthy controls, pre-cancer and cervical cancer patients. The miRNA expression level of miR-21-5p, miR-155-5p, miR-199a-5p, miR-145-5p, miR218 -5p, and miR-34a-5p in (a) urine, (b) serum, (c) tissue biopsies and (d) cervical scrape. Urine and serum samples were taken from pre-cancer and cervical cancer patients and compared to samples from healthy controls. In case of cervical scrape, the samples were derived from pre-cancer patients compared to samples from healthy volunteers, while in case of tissue biopsies, the samples were derived from cancer patient compared to samples from adjacent non-malignant tissues. **p≤ 0.01, ***p ≤ 0.001.

**Figure S2:** Correlation of expression pattern of miRNAs in cervical tissue biopsies and paired urine using Pearson’s correlation coefficient. Pearson’s correlation scatter plots for the correlation between the fold-change expression levels of (a) miR-21-5p, (b) miR-155-5p, (c) miR-199a-5p, (d) miR-145-5p, (e) miR-34a-5p, and (f) miR-218-5p in tissue biopsies and paired urine of cervical cancer patients.

**Figure S3:** Expression levels of miR-21-5p, miR-155-5p, miR-199a-5p, miR-145-5p, miR-218-5p, and miR-34a-5p in HPV16-positive and HPV16-negative urine, serum, and cervical scrape samples of cervical pre-cancer (a-c) and urine, serum, and tissue biopsies of cervical cancer patients (d-f).

**Figure S4:** (a-f) Differential expression of six miRNAs in pre-cancer, cancer and normal urine, and the ROC analyses of six miRNA detection. Box plot of miRNA expression in urine (upper: miR‐21-5p, miR‐155-5p and miR‐199a-5p; lower: miR‐145-5p, miR‐218-5p and miR‐34a-5p is normalized to U6. The lines inside the boxes indicate the medians. The boxes mark the interval between the 25th and 75th percentiles. The whiskers indicate the interval between and outside the 10th and 90th percentiles. A statistically significant difference is determined by Kruskal–Wallis test with Dunn's multiple comparison post‐hoc test, normal urine.





**Figure S5:** The target gene prediction and function analyses. The Venn diagrams represent the overlapping target genes of (a) miR-21-5p, (b) miR-155-5p, (c) miR-199a-5p, (d) miR-34a-5p, (e) miR-145-5p, and (f) miR-218-5p predicted by miRDB, Target Scan and DIANA microT‐CDS online analysis tools.

**(A) Gene Ontology Enrichment**

**(B) Pathways Enrichment Analysis**


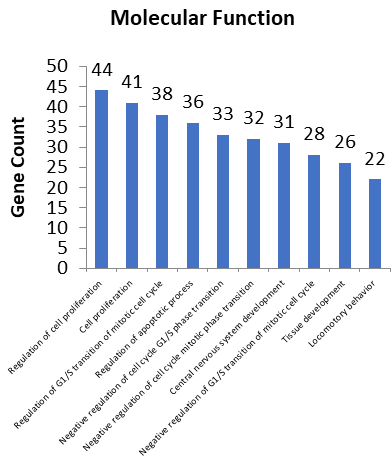

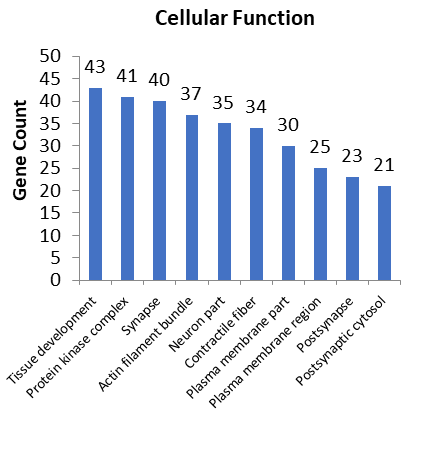

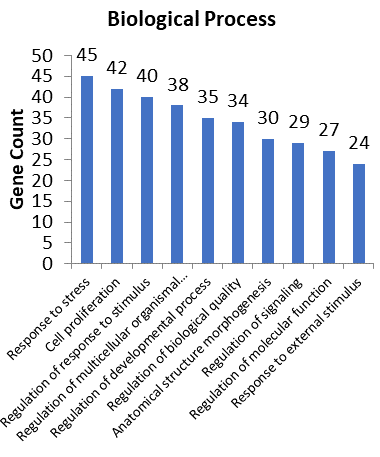


**(c)**

**(b)**

**(a)**

**Figure S6:** **A**] Top 10 Gene ontology enrichment in (a) Biological process, (b) Molecular, and (c) Cellular functions. **B**] Top 10 pathway enrichment analysis.

|  | **SiHa (HPV16 positive)** | | **HeLa (HPV18 positive)** | | **C33a (HPV negative)** | |
| --- | --- | --- | --- | --- | --- | --- |
| **miRNAs** | **FC±SD** | ***p value*** | **FC±SD** | ***p value*** | **FC±SD** | ***p value*** |
| miR-21-5p | 4.98±1.5 | <0.0001 | 2.95±1.2 | <0.0001 | 1.36±0.8 | <0.001 |
| miR-155-5p | 3.86±0.9 | <0.0001 | 2.43±2.0 | <0.0001 | 1.58±1.0 | 0.001 |
| miR-199a-5p | 3.03±0.5 | <0.0001 | 2.07±0.9 | <0.0001 | 1.09± 0.6 | 0.01 |
| miR-145-5p | 0. 18±0.6 | <0.0001 | 0.34±0.1 | <0.0001 | 0.42±0.1 | <0.0001 |
| miR-218-5p | 0. 26±0.4 | <0.0001 | 0.59±0.1 | <0.0001 | 0.65±0.4 | 0.001 |
| miR-34a-5p | 0.45±0.2 | <0.0001 | 0.87±0.2 | 0.01 | 0.75±0.1 | <0.01 |

**SUPPLEMENTARY TABLES**

**Table S1: The expression of miRNAs in HPV positive and HPV negative cervical cancer cell line**

*FC: Fold change; SD: standard deviation*

**Table S2: The expression of miRNAs according to HPV infection status in urine, serum, cervical scrape, and tissue biopsies derived from cervical pre-cancer and cancer patients**

| **Sample Types** | **Pathology** | **miR-21-5p**  **(NFE)**  **FC±SE** | ***p*** | **miR-145-5p**  **(NFE)**  **FC±SE** | ***p*** | **miR-218-5p**  **(NFE)**  **FC±SE** | ***p*** | **miR-34a-5p**  **(NFE)**  **FC±SE** | ***p*** | **miR-155-5p**  **(NFE)**  **FC±SE** | ***p*** | **miR-199-5p**  **(NFE)**  **FC±SE** | ***p*** |
| --- | --- | --- | --- | --- | --- | --- | --- | --- | --- | --- | --- | --- | --- |
| Urine  (n=75)  HPV16/18 positive | Pre-cancer  (n= 35) | 2.1±0.2 | 0.64 | 0.2±0.01 | 0.007 | 0.4±0.03 | 0.81 | 0.4±0.02 | 0.851 | 2.0±0.3 | 0.40 | 2.6±0.8 | 0.75 |
|  | Cancer  (n= 40) | 3.0±0.1 | 0.68 | 0.3±0.01 | 0.02 | 0.5±0.02 | 0.15 | 0.4±0.02 | 0.773 | 2.4±0.5 | 0.54 | 1.8±0.6 | 0.16 |
| Urine  (n=25)  HPV16/18 negative | Pre-cancer  (n= 15) | 1.8±0.4 |  | 0.6±0.02 |  | 0.5±0.03 |  | 0.6±0.05 |  | 1.7±0.2 |  | 2.2±0.2 |  |
|  | Cancer  (n= 10) | 2.2±0.3 |  | 0.5±0.02 |  | 0.6±0.01 |  | 0.4±0.1 |  | 1.5±0.3 |  | 1.7±0.6 |  |
| Serum  (n=75)  HPV16/18 positive | Pre-cancer  (n= 35) | 2.3±0.8 | 0.04 | 0.1±0.01 | 0.77 | 0.3±0.04 | 0.64 | 0.3±0.02 | 0.231 | 2.7±1.0 | 0.47 | 1.7±0.5 | 0.47 |
|  | Cancer  (n= 40) | 3.2±0.6 | 0.47 | 0.12±0.1 | 0.02 | 0.32±0.05 | 0.86 | 0.32±0.08 | 0.551 | 2.5±1.5 | 0.41 | 2.0±0.7 | 0.90 |
| Serum  (n=25)  HPV16/18 negative | Pre-cancer  (n=15) | 1.4±0.5 |  | 0.5±0.01 |  | 0.2±0.08 |  | 0.4±0.01 |  | 1.9±0.5 |  | 1.9±0.2 |  |
|  | Cancer  (n= 10) | 2.2±0.3 |  | 0.24±0.01 |  | 0.3±0.01 |  | 0.26±0.03 |  | 2.0±0.6 |  | 1.6±0.1 |  |
| Scrape  (n=35)  HPV16/18 positive | Pre-cancer  (n=35) | 2.9±0.3 | 0.86 | 0.12±0.01 | 0.28 | 0.3±0.06 | 0.33 | 0.2±0.05 | 0.876 | 2.8±1.1 | 0.31 | 2.9±0.2 | 0.57 |
| Scrape  (n=15)  HPV16/18 negative | Pre-cancer  (n=15) | 2.06±0.2 |  | 0.4±0.01 |  | 0.1±0.02 |  | 0.3±0.06 |  | 2.0±0.5 |  | 2.6±0.3 |  |
| Biopsy  (n=36)  HPV positive | Cancer  (n=36) | 3.69±0.1 | 0.01 | 0.2±0.01 | 0.53 | 0.2±0.02 | 0.93 | 0.27±0.02 | 0.98 | 2.6±0.9 | 0.001 | 2.5±0.5 | 0.01 |
| Biopsy  (n=4)  HPV negative | Cancer  (n=4) | 2.88±0.5 |  | 0.3±0.01 |  | 0.2±0.01 |  | 0.3±0.02 |  | 2.1±0.1 |  | 1.9±0.1 |  |

*FC: Fold change; SE: standard error*

**Table S3: The correlation of the expression of urine, serum and cervical scrape miRNAs with the clinicopathological parameters of cervical pre-cancer patients**

| **Variables** | **Sample Type** | **miR-21-5p**  **FC±SD** | **p value** | **miR-145-5p**  **FC±SD** | **p value** | **miR-218-5p**  **FC±SD** | **p value** | **miR-34a-5p**  **FC±SD** | **p value** | **miR-155-5p**  **FC±SD** | **p value** | **miR-199a-5p**  **FC±SD** | **p value** |
| --- | --- | --- | --- | --- | --- | --- | --- | --- | --- | --- | --- | --- | --- |
| Age (years)  >40 years  (n=24) | Urine | 2.23±1.2 | 0.01 | 0.61±0.1 | 0.01 | 0.57±0.1 | 0.02 | 0.4±0.1 | 0.06 | 2.95±0.02 | 0.002 | 2.87±1.8 | 0.001 |
|  | Serum | 2.82±1.6 | 0.01 | 0.19±0.01 | 0.03 | 0.27±0.1 | 0.0002 | 0.39±0.1 | 0.07 | 2.43±0.05 | 0.0004 | 3.27±1.9 | 0.671 |
|  | Cervical scrape | 3.58±1.3 | 0.46 | 0.12±0.01 | 0.03 | 0.25±0.1 | 0.001 | 0.21±0.02 | 0.01 | 2.03±0.1 | 0.06 | 3.74±1.5 | 0.0001 |
| <40 years  (n=26) | Urine | 1.98±0.8 |  | 0.86±0.1 |  | 0.38±0.2 |  | 0.58±0.2 |  | 2.09±0.04 |  | 2.04±0.6 |  |
|  | Serum | 2.4±1.0 |  | 0.23±0.01 |  | 0.65±0.1 |  | 0.76±0.4 |  | 2.08±0.05 |  | 3.1±1.2 |  |
|  | Cervical scrape | 3.0±0.5 |  | 0.31±0.02 |  | 0.81±0.3 |  | 0.43±0.02 |  | 1.9±0.8 |  | 2.12±0.5 |  |
| Age of marriage  <18  (n=31) | Urine | 2.84±0.2 | 0.01 | 0.5±0.2 | 0.01 | 0.49±0.1 | 0.08 | 0.38±0.01 | 0.74 | 1.71±0.09 | 0.77 | 2.8±1.6 | 0.01 |
|  | Serum | 2.23±1.1 | 0.02 | 0.1±0.01 | 0.57 | 0.26±0.1 | 0.77 | 0.25±0.01 | 0.95 | 1.54±0.6 | 0.01 | 2.38±2.0 | 0.01 |
|  | Cervical scrape | 2.1±1.6 | 0.01 | 0.16±0.03 | 0.06 | 0.31±0.1 | 0.692 | 0.41±0.01 | 0.527 | 2.1±1.7 | 0.01 | 3.81±2.3 | 0.487 |
| >18  (n=19) | Urine | 2.05±1.4 |  | 0.73±0.01 |  | 0.63±0.1 |  | 0.44±0.02 |  | 2.11±1.6 |  | 1.81±0.4 |  |
|  | Serum | 1.93±0.7 |  | 0.18±0.02 |  | 0.24± 0.1 |  | 0.4±0.01 |  | 2.47±1.9 |  | 2.13±1.0 |  |
|  | Cervical scrape | 2.43±1.8 |  | 0.09±0.01 |  | 0.26±0.2 |  | 0.23±0.03 |  | 2.37±1.0 |  | 3.98±2.1 |  |
| Parity  >5  (n=35) | Urine | 1.7±0.1 | 0.04 | 0.64±0.3 | 0.16 | 0.51±0.2 | 0.06 | 0.43±0.1 | 0.07 | 1.96±0.9 | 0.10 | 1.67±1.3 | 0.001 |
|  | Serum | 1.99±0.6 | 0.01 | 0.14±0.03 | 0.01 | 0.27±0.01 | 0.05 | 0.24±0.2 | 0.05 | 2.3±1.0 | 0.73 | 2.93±1.7 | 0.0001 |
|  | Cervical scrape | 2.24±1.3 | 0.04 | 0.15±0.01 | 0.15 | 0.3±0.1 | 0.06 | 0.22±0.1 | 0.08 | 2.09±0.7 | 0.01 | 1.71±0.8 | 0.01 |
| < 5  (n=15) | Urine | 2.34±1.9 |  | 0.59±0.2 |  | 0.65±0.2 |  | 0.35±0.01 |  | 2.02±0.5 |  | 2.55±1.8 |  |
|  | Serum | 2.22±0.9 |  | 0.25±0.3 |  | 0.21±0.02 |  | 0.38±0.1 |  | 2.27±0.9 |  | 1.91±1.6 |  |
|  | Cervical scrape | 3.08±1.9 |  | 0.12±0.02 |  | 0.23±0.01 |  | 0.33±0.1 |  | 1.37±0.5 |  | 1.49±1.2 |  |

*FC: Fold change; SD: standard deviation*

**Table S4: The correlation of the expression of urine and serum miRNAs with the clinicopathological parameters of cervical cancer patients**

| **Variables** | **Sample Type** | **miR-21-5p**  **FC±SD** | **p value** | **miR-145-5p**  **FC±SD** | **p value** | **miR-218-5p**  **FC±SD** | **p value** | **miR-34a-5p**  **FC±SD** | **P value** | **miR-155-5p**  **FC±SD** | **P value** | **miR-199a-5p**  **FC±SD** | **p value** |
| --- | --- | --- | --- | --- | --- | --- | --- | --- | --- | --- | --- | --- | --- |
| Age(years)    >40 yr  (n=23) | Urine | 2.51±0.3 | 0.09 | 0.34±0.1 | 0.4 | 0.62±0.2 | 0.001 | 0.44±0.1 | 0.10 | 2.15±1.2 | 0.01 | 1.98±0.8 | 0.28 |
|  | Serum | 3.28±0.7 | 0.001 | 0.24±0.1 | 0.05 | 0.39±0.1 | 0.02 | 0.23±0.1 | 0.06 | 2.41±1.1 | 0.001 | 2.77±1.3 | 0.01 |
|  | Tumor biopsy | 3.78±2.0 | 0.02 | 0.33±0.1 | 0.3 | 0.23±0.02 | 0.02 | 0.22±0.1 | 0.07 | 2.75±0.9 | 0.01 | 3.01±1.7 | <0.0001 |
| <40yr  (n=27) | Urine | 2.32±0.7 |  | 0.51±0.2 |  | 0.98±0.2 |  | 0.42±0.01 |  | 1.98±0.6 |  | 1.87±1.2 |  |
|  | Serum | 2.52±0.2 |  | 0.13±0.02 |  | 0.45±0.1 |  | 0.20±0.01 |  | 2.00±1.1 |  | 2.15±1.1 |  |
|  | Tumor biopsy | 3.08±1.2 |  | 0.29±0.2 |  | 0.36±0.1 |  | 0.23±0.1 |  | 2.01±1.6 |  | 1.65±0.5 |  |
| Age of marriage  >18  (n=29) | Urine | 1.9±1.2 | 0.01 | 0.69±0.3 | 0.6 | 0.56±0.2 | 0.8 | 0.72±0.3 | 0.08 | 2.34± 1.2 | 0.01 | 2.15±0.01 | 0.02 |
|  | Serum | 2.08±0.8 | 0.04 | 0.51±0.2 | 0.03 | 0.43±0.1 | 0.01 | 0.37±0.2 | 0.36 | 2.98±1.3 | 0.02 | 2.49±0.4 | 0.01 |
|  | Tumor biopsy | 3.26±2.1 | 0.001 | 0.34±0.1 | 0.01 | 0.12±0.01 | 0.58 | 0.23±0.1 | 0.10 | 3.62±1.5 | <0.0001 | 3.98±1.2 | 0.001 |
| <18  (n=21) | Urine | 2.64±1.6 |  | 0.5±0.01 |  | 0.49±0.1 |  | 0.38±0.1 |  | 3.71±1.2 |  | 3.8±0.9 |  |
|  | Serum | 2.90±1.4 |  | 0.16±0.03 |  | 0.26±0.1 |  | 0.31±0.2 |  | 4.1±0.9 |  | 4.01±0.3 |  |
|  | Tumor biopsy | 3.78±1.9 |  | 0.12±0.02 |  | 0.11±0.01 |  | 0.25±0.01 |  | 3.4±1.1 |  | 4.15±2.5 |  |
| Parity  >5  (n=36) | Urine | 2.89±1.8 | 0.1 | 0.62±0.2 | 0.8 | 0.42±0.1 | 0.5 | 0.35±0.1 | 0.08 | 2.91±0.9 | 0.0009 | 1.96±1.3 | <0.0001 |
|  | Serum | 3.2±0.9 | 0.1 | 0.12±0.02 | 0.04 | 0.31±0.1 | 0.02 | 0.29±0.1 | 0.3 | 2.74±0.4 | 0.0007 | 1.94±0.6 | <0.0001 |
|  | Tumor biopsy | 3.74±2.1 | 0.02 | 0.22±0.01 | 0.02 | 0.1±0.01 | 0.04 | 0.19±0.01 | 0.001 | 1.16±0.3 | 0.0001 | 2.53±1.1 | <0.0001 |
| <5  (n=14) | Urine | 3.01±1.9 |  | 0.58±0.3 |  | 0.38±0.1 |  | 0.39±0.2 |  | 3.53±0.3 |  | 2.98±0.7 |  |
|  | Serum | 3.5±2.3 |  | 0.16±0.2 |  | 0.46±0.2 |  | 0.27±0.3 |  | 4.01±1.1 |  | 3.7±1.5 |  |
|  | Tumor biopsy | 3.36±1.9 |  | 0. 32±0.1 |  | 0.28±0.01 |  | 0.16±0.01 |  | 3.78±1.3 |  | 4.25±2.3 |  |
| Stage  I-II  (n=22) | Urine | 2.1±0.5 | <0.0001 | 0.85±0.4 | 0.001 | 0.65±0.2 | 0.01 | 0.89±0.2 | 0.01 | 1.5±1.0 | 0.02 | 1.9±0.8 | 0.001 |
|  | Serum | 2.8±0.4 | <0.0001 | 0.54±0.2 | 0.0009 | 0.42±0.1 | 0.01 | 0.79±0.3 | 0.01 | 2.0±1.2 | <0.0001 | 2.5±1.9 | 0.001 |
|  | Tumor biopsy | 3.5±1.9 | <0.0001 | 0.11±0.01 | 0.001 | 0.16±0.04 | 0.001 | 0.12±0.01 | 0.001 | 3.0±1.5 | 0.01 | 3.7±1.8 | <0.0001 |
| III-IV  (n=28) | Urine | 3.2±1.7 |  | 0.14±0.01 |  | 0.33±0.1 |  | 0.36±0.2 |  | 1.8±0.9 |  | 2.2±0.9 |  |
|  | Serum | 3.5±1.9 |  | 0.17±0.01 |  | 0.21±0.01 |  | 0.48±0.1 |  | 2.9±0.8 |  | 1.9 ±1.0 |  |
|  | Tumor biopsy | 5.3±2.5 |  | 0.01±0.002 |  | 0.18±0.02 |  | 0.11±0.01 |  | 3.40±1.8 |  | 4.23±2.1 |  |

*FC: Fold change; SD: standard deviation*

**Table S5: Primers for amplification of human papilloma virus gene sequence**

| **S. No.** | **Description** | **Primer Sequence** |
| --- | --- | --- |
| 1. | Beta – Globin | FORWARD - 5’-GAAGAGCCAAGGACAGGTAC-3’  REVERSE - 5’-CAACTTCATCCACGTTACACC-3’ |
| 2. | HPV L1 | MY09 - 5’-GCMCAGGGWCATAAYAATGG-3’  MY11- 5’-CGTCCMARRGGAWACTGATC-3’ |
| 3. | HPV 16 | FORWARD – 5’-AAGGCCAACTAAATGTCAC-3’  REVERSE - 5’-CTGCTTTTATACTAACCGG-3’ |
| 4. | HPV 18 | FORWARD - 5’-TGAGGTACCATTGGATATTT-3’  REVERSE - 5’-TAGCAAAAAGCTGCTTCACGC-3’ |
| 5. | HPV 6 | FORWARD- 5’-TAGTGGGCCTATGGCTCGTC-3’  REVERSE - 5’-TCCATTAGCCTCCACGGGTG-3’ |
| 6. | HPV 11 | FORWARD - 5’-CGAGCAGACGTCCGTCCTCG-3’  REVERSE - 5’-GGAATACATGCGCCATGTGG-3’ |

Key to Symbols: R=A+G, Y=C+T, M=A+C, K=G+T, S=G+C, W=A+T, H=A+T+C, B=G+T+C, D=G+A+T, N=A+C+G+T, V=G+A+C.

**Table S6: miRNA primer sequences used for Real-time PCR**

| **S.No.** | **Genes** | **Stem Loop Sequence for RT** | **qPCR Forward Primer** | **qPCR Reverse Primer** |
| --- | --- | --- | --- | --- |
| 1 | U6 | GTCGTATCCAGTGCAGGGTCCGAGGTATTCGCACTGGATACGACAAAATA | GCTTCGGCAGCACATATACTAAAAT | CGCTTCACGAATTTGCGTGTCAT |
| 2 | miR145-5p | ACTTCGCAACTACCGTTTGAAGGGATTC | TCG GTC CAG TTT TCC CAG | ′TGAACTTCGCAACTACCGTTTG |
| 3 | miR218 -5p | GGTCGAACGCCTAACGTCACATGGTTAG | CGAGTGCATTTGTGCTTGATCTA | TAATGGTCGAACGCCTAACGTC |
| 4 | miR34a -5p | GCAGCGAATCCACGATTAGAACAACCAG | GAATGGTGGCAGTGTCTTAGC | GGAGCAGCGAATCCACGATTAG |
| 5 | miR21-5p | CACTGTCTAGCACGACACTAATCAACATCAG | GCCCGCTAGCTTATCAGACTGATG | CAGTGCAGGGTCCGAGGT |
| 6 | miR199a-5p | GTCAGAAGGAATGATGCACAGCCAACA | ACAACCTGCGTAGGTAGTTTCATGT | CGTCAGAAGGAATGATGCACAG |
| 7 | miR155-5p | CCAGAAACCGATCAGAGTGTCCCCTATCA | CGCCATGTTTAATGCTAATCGTGA | TTCCAGAAACCGATCAGAGTGT |
